# A Maverick-like cluster in the genome of a pathogenic, moderately virulent strain of *Gallibacterium anatis*, ESV200, a transient biofilm producer

**DOI:** 10.3389/fmicb.2023.1084766

**Published:** 2023-01-26

**Authors:** Patricia Sanchez-Alonso, Elena Cobos-Justo, Miguel Angel Avalos-Rangel, Lucía López-Reyes, Gloria Luz Paniagua-Contreras, Felipe Vaca-Paniagua, Estela Anastacio-Marcelino, Ana Jaqueline López-Ochoa, Victor M. Pérez Marquez, Erasmo Negrete-Abascal, Candelario Vázquez-Cruz

**Affiliations:** ^1^Centro de Investigaciones en Ciencias Microbiológicas, Instituto de Ciencias, Benemérita Universidad Autónoma de Puebla, Puebla, Mexico; ^2^Carrera de Biología, Facultad de Estudios Superiores de Iztacala, UNAM, Los Reyes Iztacala, Estado de, México, Mexico; ^3^Subdirección de Investigación Basica, Instituto Nacional de Cancerología, CDMX, México; ^4^Diagnóstico y Patobiología Aviar, Biotecnología Veterinaria S.A.-Biovetsa, BIOVETSA, Tehuacán, Mexico

**Keywords:** genome, fimbriae, biofilm, pathogenesis, avian diseases, *Gallibacterium anatis*, Maverick genetic element

## Abstract

**Introduction:**

*Gallibacterium anatis* causes gallibacteriosis in birds. These bacteria produce biofilms and secrete several fimbrial appendages as tools to cause disease in animals. *G. anatis* strains contain up to three types of fimbriae. *Complete genome sequencing* is the strategy currently used to determine variations in the gene content of *G. anatis*, although today only the completely circularized genome of *G. anatis* UMN179 is available.

**Methods:**

The appearance of growth of various strains of *G. anatis* in liquid culture medium was studied. Biofilm production and how the amount of biofilm was affected by DNase, Proteinase K, and Pronase E enzymes were analyzed. Fimbrial gene expression was performed by protein analysis and qRT-PCR. In an avian model, the pathogenesis generated by the strains *G. anatis* ESV200 and 12656-12 was investigated. Using bioinformatic tools, the complete genome of G. anatis ESV200 was comparatively studied to search for virulence factors that would help explain the pathogenic behavior of this strain.

**Results and Discussion:**

*G. anatis* ESV200 strain differs from the 12656-12 strain because it produces a biofilm at 20%. *G. anatis* ESV200 strain express fimbrial genes and produces biofilm but with a different structure than that observed for strain 12656-12. ESV200 and 12656-12 strains are pathogenic for chickens, although the latter is the most virulent. Here, we show that the complete genome of the ESV200 strain is similar to that of the UNM179 strain. However, these strains have evolved with many structural rearrangements; the most striking chromosomal arrangement is a Maverick-like element present in the ESV200 strain.

## 1. Introduction

*Gallibacterium anatis* is a gram-negative, pleomorphic and capsulated gammaproteobacterium that lacks flagellar motility. The genus *Gallibacterium* was taxonomically classified as a new member of the *Pasteurellaceae* Pohl family in 2003 by Christensen et al. by reclassification of some biovars from *Pasteurella haemolytica*, “*Actinobacillus salpingitidis*” or *Pasteurella anatis* using pulsed-field gel electrophoresis (PFGE), amplified fragment length polymorphism (AFLP), and the amplification patterns of the 16S rRNA, 23S rRNA, and ITS sequences (Christensen et al., 2003; Bojesen et al., 2007). *G. anatis* has been isolated from the reproductive and upper respiratory organs of healthy birds as part of the microbiota (Bisgaard, 1977). It has also been isolated in pure culture form and found to be associated with pathogens such as avian pathogenic *Escherichia coli* (APEC) in clinical cases of salpingitis, peritonitis, hepatitis, and septicemia in birds (Neubauer et al., 2009). These findings have led to controversy regarding its pathogenic character and its potential to cause pathogenic lesions that affect reproductive organs and egg laying in farm chickens (Bojesen et al., 2003, 2004).

However, to date, *G. anatis* has been studied as an opportunistic pathogen that can colonize chickens at 4 days of age and persist until they grow into laying hens (Huangfu et al., 2012). The detrimental effects of this genus on chicken breeding have enhanced its importance as an economic risk, and hence, specific tests to unequivocally identify *Gallibacterium* species have been sought and reported (Bojesen and Shivaprasad, 2007; Alispahic et al., 2011).

The bacterium *G. anatis* possesses several virulence factors, such as the toxin GtxA, capsule, metalloproteases (Garcia-Gomez et al., 2005), hemagglutinins, outer membrane proteins and vesicles (Chantes-Guerra et al., 2022), biofilm production genes, and gene clusters that encode F17-like fimbriae (Kudirkiene et al., 2014; Pors et al., 2016), similar to those frequently found in several pathotypes of *E. coli*, including APEC (Bager et al., 2013a; Persson and Bojesen, 2015; Bertelli et al., 2017). Among these, the toxin GtxA, which is similar to HlyA from *E. coli*, is usually identified in wild isolates, as it enables the pathogen to cause cell damage and induce apoptosis, and the presence of this toxin can be used to classify strains as hemolytic and nonhemolytic in blood agar tests (Kristensen et al., 2011).

Biofilms represent consortium-style microbial growth in which individuals of the same or different species grow embedded in a matrix composed of exopolysaccharides, proteins and deoxyribonucleic acid (DNA) (Donlan, 2002; Di Martino, 2016). Data from de Oliveira-Garcia et al. (2003) suggest that F17-like fimbriae could be important for bacterial adhesion to mammalian cells, hemagglutination, and biofilm formation. Similar data have been reported by Wurpel et al. (2016), who found that fimbriae are involved in biofilm formation and adherence to human cells. Interesting data are obtained when comparing natural bacterial isolates; a majority of *G. anatis* strains produce filaments arranged as latices and form biofilms, exhibiting adhesion to cells and inert surfaces (Vaca et al., 2011; Salgado Lucio et al., 2012), and it has been proposed that some *G. anatis* lineages carry different F17-like fimbriae and are more virulent (Bager et al., 2013b; Pors et al., 2016). The molecular classification of the F17-like genes into operons was performed by Kudirkiene et al. (2014). If biofilms are important for attachment to and colonization of animal tissues by pathogenic bacteria, strains deficient in biofilm formation could exhibit altered virulence. The goal of this research was to investigate the participation of F17-like fimbriae in agglutination, biofilm formation, and pathogenesis and to determine the full genome sequence of the *G. anatis* strain ESV200 showing a dispersible phenotype.

## 2. Materials and methods

### 2.1. Strains and media

The *G. anatis* strains used in this work were 12656-12 (β-hemolytic biovar, isolated from chicken liver by Bojesen et al., 2003) and ESV200 (β-hemolytic, isolated in Tepatitlan, Jalisco, Mexico (geographical coordinates: 20° 49′ 0″ north, 102° 44′ 0″ west), in 2009 from hens with respiratory disease by Dr. Edgardo Soriano). Strain purity was checked by streaking on blood agar medium with incubation at 37°C for 24 h. For growth in liquid medium, strains were grown at 37°C for 24 h in brain-heart infusion (BHI) medium (Merck®) with aeration, unless specified otherwise. Precultures were incubated until the optical density at 595 nm (OD_595_) was 1.0 before inoculation in BHI broth in either flasks or microplates (Chantes-Guerra et al., 2022).

### 2.2. Biofilm formation

Biofilm formation assays were performed as described by Johnson et al. (2013), with some changes as described here, using 24-well cell-culture microplates (TrueLine, LabSource Northlake, IL, United States). For the first inoculum, *G. anatis* strains were grown in tubes containing 5 ml of liquid BHI medium at 37°C for 18 h with aeration. Bacterial cells were concentrated by centrifugation and adjusted to an OD_600_ of 2.0, and 1:100 dilutions of each strain in liquid BHI medium were prepared. For each dilution, five wells were filled with 1.5 ml of medium, and four of the 24 wells on the plate were reserved for the control, which consisted of BHI inoculated with phosphate-buffered saline (PBS) as described by Cramton et al. (1999). The subsequent assays were performed in triplicate, and the plates were incubated at 37°C under static conditions for 24, 48, 72, and 96 h. After each incubation period, the culture medium and planktonic cells were removed carefully from the wells, and each well was washed twice with triple-distilled water to avoid shearing the biofilm mechanically *via* water pressure or contact with the pipette or removal of the biofilm from the surface. The plates with biofilms were completely dried by placing them upside down on blotting paper, and each well was filled with 1.5 ml of 0.1% violet crystal, after which the plates were allowed to stand for 30 min at room temperature. The wells were washed four times as described above and dried at room temperature for approximately 45 to 60 min. Two milliliters of acetic acid (33% v/v) were added to each well to solubilize the crystal violet, which was quantified by measuring the OD_630_ in disposable polystyrene cuvettes. To avoid variations in the analysis due to the volatility of acetic acid, the solubilization procedure was performed in five sample blocks in one row at a time to prevent acetic acid evaporation. Normalization of the biofilm data was performed using duplicates of each treatment and each replicate. For each cell culture in the plate used for biofilm quantification, another plate was used for cell quantification by resuspending the biofilms, homogenizing and measuring the OD_600._

For protein analysis, biofilms were recovered from large-volume cultures of *G. anatis* strains. For this purpose, 150 ml of BHI medium was used to dilute the standardized inoculum at 1:100 as described above. The inoculated medium was placed in 1 L glass bottles placed laying on their sides, and the bottles were incubated statically at 37°C for 24, 48, 72 and 96 h. The medium and planktonic culture were removed carefully by decantation. To recover the biofilms, 2 ml of 50 mM Tris buffer (pH 7.5) was added, and glass beads were used to release the biofilm layer, which was recovered in 15 ml Falcon tubes. The remaining residues were recovered with 500 μl of 50 mM Tris buffer (pH 7.5). The samples were stored at-80°C. The recovered biofilms were subjected to a complex analysis of the changes in their absorbance and protein profiles after treatment with 4 M urea for 30 min, DNase (100 μg/ml), lysozyme (50 μg/ml), proteinase K (50 μg/ml), and pronase E (100 μg/ml). Denaturing 10% polyacrylamide gel electrophoresis (SDS–PAGE) of the biofilm proteins was performed to explore and distinguish planktonic cell growth from adherent cell growth, as reported previously (Lopez-Ochoa et al., 2017).

### 2.3. Nucleic acid manipulation

Total DNA from *G. anatis* was obtained from broth cultures grown at 37°C for 24 h. A total of 1.5 ml of the culture was centrifuged at 13,000 rpm for 5 min; the cell pellet was recovered, 0.5 ml of lysis buffer (50 mM Tris–HCl (pH 8.0), 10 mM EDTA, 10 mM NaCl) was added, and the sample was homogenized. Then, 20 mg of lysozyme were added and the sample was incubated at 37°C for 20 min; afterward, 40 μl of 1% sodium dodecyl sulfate (SDS) were added, and the sample was homogenized. The lysate was treated with 200 μl of phenol saturated with a solution of Tris and EDTA (or TE) (Sambrook and Russell, 2001), stirred and centrifuged at 13,000 rpm for 10 min. One volume of phenol-TE/chloroform/isoamyl alcohol (25:24:1, v/v) was added to the aqueous phase, and the sample was homogenized and centrifuged at 13,000 rpm for 10 min. The supernatant was recovered, washed twice with chloroform, and centrifuged at 13,000 rpm for 5 min; the upper phase was recovered in a new tube, either 0.1 volume of 3 M sodium acetate (pH 7) or 0.5 volumes of 7.5 M ammonium acetate (pH 7) was added, and the sample was homogenized. Total DNA was precipitated with cold (−20°C) 70% ethanol in water. The mixture was centrifuged at 13,000 rpm for 10 min, the supernatant was discarded, and the DNA pellet was washed with 1 ml of cold 70% ethanol. The DNA was dissolved in 100 μl of sterile distilled water and stored at −20°C until use.

Ribonucleic acid (RNA) was extracted from *G. anatis* under two different growth conditions, namely, agitation or static conditions, in which the OD_600_ was 1 and 0.5, respectively. To obtain *G. anatis* ESV200 RNA, a QIAcube Connect (Qiagen® Cat. No./ID: 9002864) was used. In each case, 20 ml of the bacterial culture was centrifuged at 3,500 rpm for 10 min. The supernatant was discarded, and the excess broth was removed with a pipette tip. Then, 500 μl of lysis buffer (10 mM Tris–HCl (pH 8.5), 10 mM EDTA, 10 mM NaCl) was added, and the mixture was homogenized. Then, 500 μl of acid-chloroform-isoamyl phenol was added, and the sample was shaken manually for 2 min and centrifuged at 3,500 rpm for 10 min in a table top centrifuge. The supernatant was transferred to another tube, and 200 μl of 7.5 M ammonium acetate and 500 μl of acid-chloroform-isoamyl phenol were added. The sample was then mixed vigorously for 2 min before centrifugation at 3,500 rpm for 10 min. The aqueous phase was transferred to another tube and washed twice with chloroform. After centrifugation, the aqueous phase was precipitated with 2 volumes of cold absolute ethanol and allowed to precipitate overnight. To recover, the RNA mixture was centrifuged at 3,500 rpm for 10 min, and the pellet was washed twice with cold 70% ethanol. Once dry, the pellet was dissolved in 200 μl of diethyl pyrocarbonate (DEPC)-treated water. The nucleic acid purity was estimated by spectrophotometry based on its absorbance at 260, 230, and 280 nm; RNA integrity was checked by analyzing the electrophoretic profile in a 1% agarose gel, and the samples were stored at −80°C until use.

### 2.4. Pathogenicity tests

Bacteria were cultivated on 10% sheep blood agar at 37°C and incubated overnight in a candle jar. The bacterial inoculum was prepared by cultivation of each strain for 14 h in BHI broth.

Specific pathogen-free chickens (Alpes I, Puebla, México) of mixed sexes were divided into two groups of 5 chickens each. The chickens were individually labeled and injected intravenously with 0.2 ml of *G. anatis* inoculum (approximately 1 × 10^8^ viable cells). The negative control group was inoculated by intravenously injecting 0.2 ml of BHI medium.

Clinical examination, morbidity and mortality data were recorded for each bird group at 7 days post-infection (dpi). Subsequently, the animals were euthanized, and the skin and mucosa (crest, chin, nostrils, eyes, skin, eyelids), respiratory system (infraorbital sinuses, trachea, syrinx, lungs), digestive system (oral cavity, esophagus, craw, proventriculus, gizzard, liver, pancreas, duodenum, jejunum and ileum, blind, rectum, tonsils C, palatal fissure), circulatory and hemolymphatic apparatus (heart, spleen, pouch of Fabrizio, thymus), genitourinary apparatus (testis, ovary, oviduct, follicle, kidneys, ureters), and skeletal muscle system (bones, joints, plantar tissue, bone marrow) from all birds were examined for histopathological signs, followed by bacterial isolation. Samples of tissues with lesions were collected with sterilized cotton swabs, streaked on blood agar plates, and incubated for 24 h at 37.5°C in a microaerophilic environment.

For histopathological analysis, the samples were placed in 10% neutral buffered formalin and processed, sectioned, and stained as described elsewhere. Microscopic lesions were scored on a scale of 0–3: 0 = normal, 1 = minimal changes, 2 = mild changes, and 3 = severe changes. When the observation fields of a single section had lesions with different severities, the lesion degree predominant in 50% or more of the observation fields was used for the aforementioned scoring. The average score for each group was determined by adding individual scores and dividing the total score by the number of slides examined.

The Kruskal–Wallis test was used to assess the differences in the median scores of microscopic lesions between the groups, and differences were considered significant at *p* = 0.05, as reported elsewhere. Chi-square tests were used to examine the differences between the groups in isolation (presence of the inoculated strains in either sinus) and morbidity rates (percentage of birds that showed any clinical signs during the 7-day observation period), with differences being considered significant at *p* = 0.05 (Zepeda-Lopez et al., 2010).

### 2.5. Reverse transcription and end-point polymerase chain reaction

To analyze the changes in the transcriptional expression of the fimbrial and adhesin genes of both the 12656-12 and ESV200 strains by end-point RT–PCR, we used the UMN179 genome sequences (http://www.ncbi.nlm.nih.gov/genome/3653?project_id=66567 from Johnson et al., 2011) of the gene Flf1_a to design the primer pair Fifa7upp and Fifa7rev that amplified a 236-bp fragment (Table 1). The genome sequences of the 12656-12 strain^1^ were used for subsequent primer design: for the Flf3_b, Flf_b, Flf3, Flf1, and, Flf-related genes, pairs of primers were designed, whose names, primer sequences and sizes of the PCR amplicons are shown in Table 1. It was not necessary to make more primer designs because the size of the synthesized amplicons was appropriate to carry out the end-point PCR and qRT–PCR (see below). As a positive control, total DNA from *G. anatis* 12656-12 was used for PCR, and as a negative control, a reaction without reverse transcriptase was performed.

**Table 1:** Oligonucleotide primers used for end point RT–PCR and qRT–PCR.

Gene	Primer name	F/R*	Sequence (5’to 3′)	Amplicon size (bp)
*flf1_a*	Fifa7upp	F	AATCAACTTTGTCTGGGGTAGGA	236
	Fifa7rev	R	TGCTGCAGATAATTCAAAAGACG	
*flf3_b*	FifAOPdir	F	ATGGTGCTTTTGCTAGTGGTGAA	281
	FifAOPrev	R	AGGCTCCTTGTCTGCATAATAACG	
*flf_b*	FifARTup	F	CTGGTGATGCAGCTCAAAATG	141
	FifARTrev	R	CAATGTGGCTGTTTTCTTGC	
*flf3_D*	Adhlike31TRDir	F	TCGATGATGGCTTTGGTGA	223
	Adhlike31TRRev	R	CCGTTGGATTCGTTACA	
*flf1_D*	FifG7Dir	F	CAGGTCTACAAATTACGCCAAG	147
	Fifg7Rev	R	CGTGCTTGCATCAGAAAAGG	
*flf_D-*related	22AdhlikeTREDir	F	GGTAAATCTCGCCCCTGTT	189
	22AdhlikeTRERev	R	TTTCTTTTAGTACTACCGTTCTGG	
*rpoA*	rnapolMdir	F	TACCAACAAACGACCGATAGG	244
	rnapolMrev	R	TAAAGTGCAGAATAAAGATGACG	
*fusA*	EfactorGFMdir	F	TGAATGCCAATGCTGAGAATG	248
	EfactorGFMrev	R	TGGCGGTGAAGAACTAACTG	

* F/R indicates the forward or reverse primer for each gene target.

The reaction mixture for endpoint PCR contained 10 ng of total DNA from *Gallibacterium* strains as template, 0.3 μM each DNA oligonucleotide primer, 10 mM Tris–HCl (pH 8.4), 1.5 mM MgCl_2_, 0.24 mM each dNTP and 0.3 U of Platinum *Taq*DNA polymerase (Invitrogen, Thermo Fisher Scientific. Waltham, MA, United States). The mixture was homogenized and treated as follows: denaturation for 5 min at 95°C, followed by 30 cycles of the appropriate annealing temperature and 72°C for elongation in a T100 thermal cycler (Bio-Rad, Hercules, CA, USA). The amplified products were electrophoresed in a 0.8% agarose gel, stained with ethidium bromide solution (2 μg/ml) and observed under ultraviolet light.

### 2.6. Quantitative and end-point PCR

To analyze the changes in transcriptional expression by RT–PCR, the Ct cycle method was used. Using the constitutively expressed genes as a reference, we chose the hypothetical 901-bp *rpo*A gene, which encodes the RNA-polymerase alpha-subunit RpoA, as well as the 2,106-bp putative *fus*A gene from *G. anatis* UMN179, which encodes a hypothetical elongation factor G (EFG). The primers for these two genes and the primers used for analysis of the expression of the fimbrial genes are listed in Table 1. Due to the small size of the amplicons and the fact that the primers were well evaluated for their amplification efficiency, the same primers were used in the end-point PCR and quantitative RT-PCR. We expected that the transcripts of the housekeeping genes would show stable levels of expression under different conditions for normalization, and we chose to apply the formula described by Pfaffl (2001).

Real-time PCR was performed in an IQTM5 Multicolor Real Time PCR Detection System (Bio-Rad). For each qPCR sample, three technical replicates and two biological replicates were examined. For each primer pair, a no-template control (NTC) was included to detect the formation of primer dimers. The reaction mixture was prepared at room temperature as follows: 1 μl of cDNA, 1 μl of 10 μM forward primer, 1 μl of 10 μM reverse primer, 10 μl of SYBR Green PCR master mix (Thermo Scientific) and 7 μl of water. The SYBR Green mix already contains DNA polymerase, SYBR Green and a passive reference dye (ROX), along with the required reaction components. Individual concave-cap tubes were used. The reaction was carried out according to the supplier’s instructions: 1 cycle of 95°C/10 min; 40 cycles of 95°C/15 s, 60°C/15 s; 72°C/30 s. For the denaturation curve, 60 cycles at 65°C were performed, with the time increasing by 5 s in each cycle. Once the experimental Ct values were obtained, the dynamic range was calculated using log_10_ dilutions for the test, covering a range of 10^0^ to 10^8^ molecules. The efficiency of the qRT-PCRs was determined, and those with efficiencies greater than 96% and an R^2^ value greater than 0.86 were considered valid. The Supplementary material includes graphs showing the expression of some genes.

### 2.7. Genomic analysis of Gallibacterium anatis ESV200

The total DNA of *G. anatis* ESV200 was obtained to carry out nucleotide sequencing of the genome. Two sequencing technologies, namely, the NextSeq500 platform from Illumina® and the Sequel System from PacBio®, were used to obtain a unique chromosomal molecule. The software packages used to join reads into a single chromosomal sequence were FASTQC 11.9 (Wingett and Andrews, 2018), Velvet 1.0.19 (Zerbino and Birney, 2008), Spades 3.15.5 (Bankevich et al., 2012), and Canu 2.3 (Koren et al., 2017). The gene content in the genome of the ESV200 strain was explored by comparing the information deposited in GenBank under accession number CP114281 (WGS- Project-CP114281.1) and three other records for strains UMN179 (CP002667), F149 (JPHN01000000) and 12656-12 (AVOX00000000). The genomes included correspond to the most well-studied strains of *G. anatis*, and because the pathogenicity was compared with reference to the virulent strain 12656-12, UMN179 is the only completely circularized genome. Complete chromosomes were compared (Dhillon et al., 2013) with *R*apid *A*nnotation using *S*ubsystem *T*echnology (RAST; Aziz et al., 2008; Overbeek et al., 2014) and displayed in a graphical representation prepared with the Easyfig program (Sullivan et al., 2011). Additionally, a review of the gene annotation obtained by the GenBank pipeline was performed. The contents of various RNA types were verified using *B*asic *L*ocal *A*lignment *S*earch *T*ool - BLASTn (www.ncbi.nlm.nih.gov; Madden et al., 1996) and tRNAscan-SE 2.0 (Chan et al., 2021). The phylogenetic analysis of the four fimbrial operon genes was performed as described Kudirkiene et al. (2014), although the sequences of the genes encoding the Usher chaperone, the major protein, and the adhesin were separated for a more appropriate comparison. Clusters were constructed by comparison of *G. anatis* gene sequences from operons with Clustal Omega (Madeira et al., 2022): the sequences included here were from the *G. anatis* ESV200, UMN179, and 12656-12 strains. The phylogenetic analysis of *G. anatis* ESV200 was performed by comparing eight concatenated housekeeping genes (*adk, atp*D, *fum*D, *gyr*B, *inf*2, *mdh*, *rec*N, and *tdh*) previously used as *m*ultilocus *s*equence *t*yping (MLST) markers in *G. anatis* (Johnson et al., 2013). The neighbor-joining method was used to draw the dendrogram using the online programs Clustal Omega,^2^ Kalign (Lassmann, 2019) and iTol (Letunic and Bork, 2021). A phylogram was constructed with or without a root of two MLST sequences from *Avibacterium endocarditis* 20186H4H1 and *A. gallinarum* NCTC11188 strains (Accession numbers: NZ_PQVI01000000 and NZ_PQVJ00000000.1, respectively). MEGA v.11 software was used to assess the confidence of the phylogram of *G. anatis* MLST sequences through a bootstrapping test with 500 resamples.

## 3. Results

### 3.1. Gallibacterium anatis growth and biofilm production

**Figure 1:**
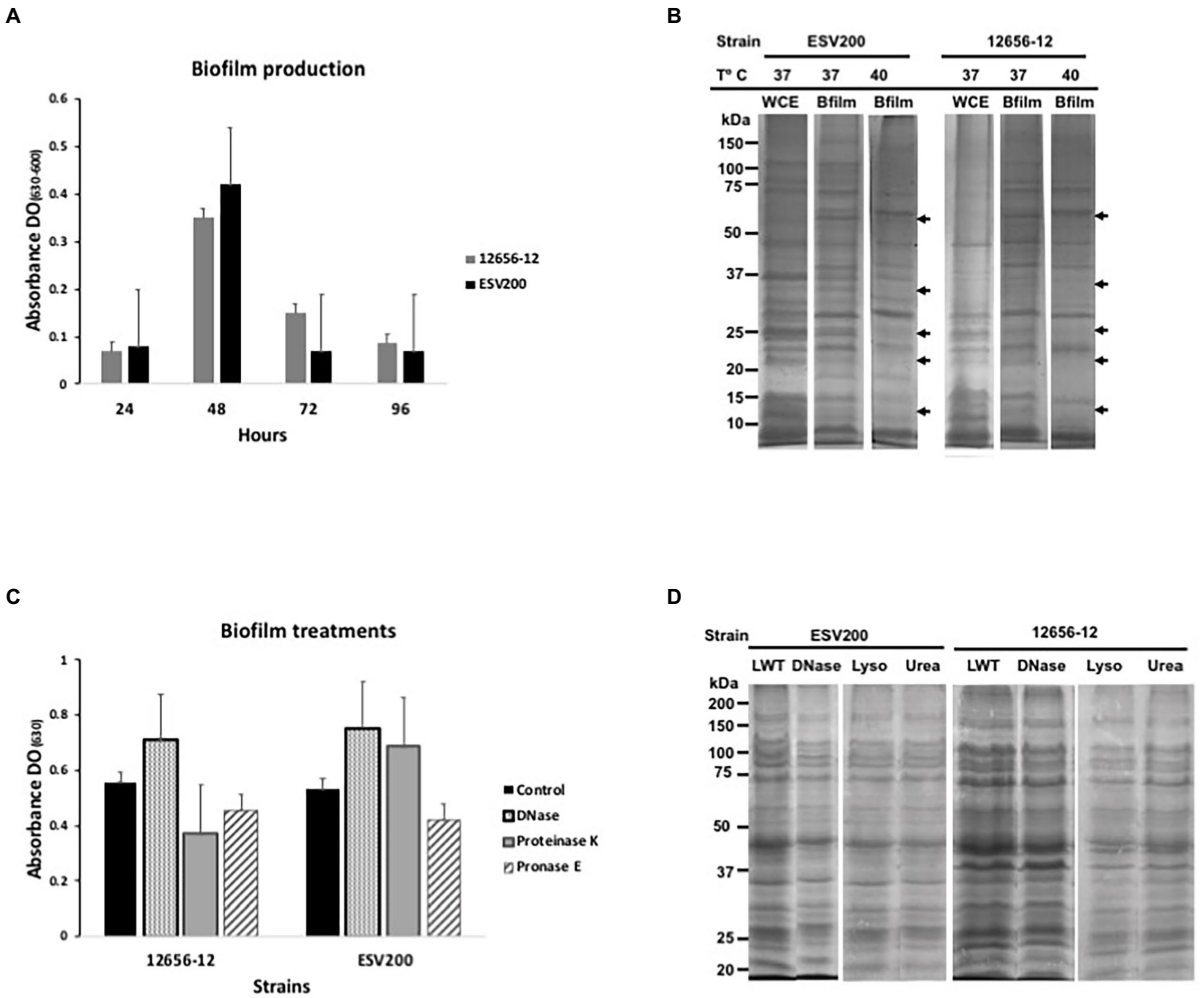
Biofilm production and protein expression pattern from *G. anatis* 12656-12 and ESV200 strains cultured under different conditions and subjected to treatment. **(A)** Biofilm production analysis as a function of culture incubation time (normalized); the optical density (OD_630_) corresponds to the absorbance of the crystal violet dye retained by the biofilm. **(B)** Protein profiles from biofilms produced by *G. anatis* cultured at different temperatures. The arrows indicate the disappearance of protein bands at high temperature. WCE-whole cell extract. **(C)** Alteration of the enzyme-treated *G. anatis* biofilm produced in wells of polystyrene plates (as described in the Materials and methods); the optical density (OD_630_) corresponds to the absorbance of the crystal violet dye retained by the biofilm after treatment. **(D)** Protein profiles of the *G. anatis* biofilms harvested and previously treated with DNase, lysozyme, or urea before separation by denaturing electrophoresis (SDS–PAGE; as described in the Materials and methods). LWT, left without treatment.

Usually, *G. anatis* 12656-12 and ESV200 strains cultured in the same conditions show different turbidity or clumping phenotypes in liquid BHI medium, and 12656-12 strain cultures agglutinate after a few minutes if kept under static conditions; on the other hand, ESV200 strain cultures form stable dispersed suspensions, and this behavior could be a result of inoculum size, aeration and biofilm capacity. To analyze the development of turbidity and cell agglomeration, two types of cultures were prepared by incubation at 37°C, one agitated at 200 rpm to force the formation of a homogeneous suspension and the other unagitated. The shaken cultures consistently exhibited an OD_600_ of 1.9 at 24 h, while the static cultures showed a maximal OD_600_ of 1.9 at 96 h for *G. anatis* 12656-12 and a maximal OD_600_ of 1.2 at 24 h for *G. anatis* ESV200. Two conditions were established for comparative quantitation of gene expression in *G. anatis* at different culture times, as shown below. The results for the quantitation of biofilm formation are depicted in Figure 1, showing an increase in biofilm quantity for both strains at 48 vs. 24, 72, and 96 h. As depicted in Figure 1A, strain ESV200 showed maximal production of biofilm at 48 h, which is 25% more than the amount of biofilm produced by the 12656-12 strain. However, the ESV200 biofilm was unstable, showing a drastic decrease of approximately 80% between 48 and 72 h. The homogeneous suspension and the minor quantity of biofilm formed confirmed the predominant growth of ESV200 in liquid culture medium as a major planktonic suspension, with a temporary exception at 48 h. In contrast, the 12656-12 strain was clumpy in liquid BHI medium and maintained the biofilm structure for a long time until the cell density was double that of ESV200 at 72 h. These differences observed for the ESV200 strain cultures indicated that biofilm formation was turned on in a transient manner after disintegration of the biofilm structure but did not indicate an inability to produce a biofilm. The 12656-12 strain showed a robust biofilm.

### 3.2. Protein production analysis of the *Gallibacterium anatis* ESV200 and 12656-12 strains

The protein profiles of the planktonic and biofilm cultures were studied using SDS–PAGE to determine the importance of this type of cellular component in the formation of aggregates or in maintaining cellular dispersion. The protein patterns of bacterial growth of both strains under agitation showed higher levels of recoverable and visible proteins in the SDS–PAGE with differences in the levels of some proteins, which suggests that the proteins could be relevant in the adherence or dispersibility of both strains, phenotypes that differed between these two strains (Figure 1B). Therefore, it can be inferred that the ESV200 strain, because of its proteins, was more compatible with the aqueous medium because it remained dispersed for a longer time. Cultures of the same strain with planktonic cells and biofilms also showed differences in the expression patterns of different proteins. The ESV200 strain showed five protein bands with increasing concentrations in the biofilm structure at 37°C. The concentrations of the visible proteins decreased at 42°C, indicating regulation mediated by temperature.

In *G. anatis* 12656-12, an increase in the levels of more than 6 accumulated protein bands in the adherent state was observed (the arrows show the 5 most evident bands of less than 100 kDa). This profile was maintained at 37 or 42°C of incubation; therefore, this variation in the culture conditions led to little alteration in 12656-12 strain metabolism.

As it has been reported that bacterial biofilms represent a state of cell agglomeration with a complex matrix, the biofilm was treated for 48 h with 4 different enzymes (two proteases, DNase and lysozyme) and urea (Figures 1C,D) to explore the changes in protein profile in the biofilm matrix. These treatments would increase the turbidity of the biofilm suspension if they separated the cell clumps. Figure 1C shows the change in cell agglomeration caused by DNase, proteinase K and pronase E. In samples from the ESV200 biofilm, DNase and proteinase K treatments increased the turbidity measured at 630 nm, while pronase E reduced the turbidity. For the 12656-12 biofilm, only treatment with DNase disintegrated the agglomerates and induced an increase in the OD; the proteases used did not have this effect. The protein profile was not altered by DNase, lysozyme or urea, although the separation of the proteins was slightly improved with respect to the profile of the proteins obtained by simple resuspension in the 2X loading buffer used for SDS–PAGE.

### 3.3. Pathogenicity test of *Gallibacterium anatis* ESV200

Because the two strains of *G. anatis* investigated had different agglomeration behaviors, their pathogenic capacity was analyzed in groups of five birds at 8 weeks of age, and relatively similar results were obtained when the birds were inoculated intravenously with 0.2 ml of fresh culture. The symptoms appeared on the 4th day postinoculation, although there was greater decay in the group inoculated with ESV200 (Table 2). There was no evident damage to the respiratory system of birds inoculated with any of the strains during necropsy. However, a higher rate of damage was observed in the organs (liver, spleen or kidney) of the birds inoculated. Damage to the heart was observed in birds inoculated with the *G. anatis* 12656-12 strain but not in birds inoculated with the ESV200 strain. The injected bacteria were unable to be recovered from the most frequently damaged organs.

**Table 2:** *Gallibacterium anatis* pathogenesis test.

Experimental infectious disease assay		Data assay		
*G. anatis* strain		Control (−)	12656-12*	ESV200**
Infection route		intravenous	intravenous	intravenous
Number of chickens by group		5	5	5
Chicken age (weeks old)		8	8	8
Symptoms appearance time (dpi)		none	3	3
External signs	Decayed (3dpi)	none	5/5	5/5
	Limp combs	none	2/5	5/5
Euthanasia and necropsy (dpi)		7	7	7
Pathological data observed				
	Respiratory tract damage	absent	absent	absent
	Liver congestion	none	3/5	3/5
	Liver atrophy	none	1/5	none
	Heart hydropericardium	none	1/5	none
	Hydropericardium onset	none	3/5	none
	Splenomegaly***	none	3/5	5/5
	Spleen atrophy	none	1/5	none
	Spleen congestion	none	3/5	3/5
	Kidneys	none	5/5	3/5
Bacterial recovery from organs				
	Spleen	Not tested	Negative	Negative
	Liver	Not tested	Negative	Negative

The potential pathogenicity of the *G. anatis* 12656-12 and ESV200 strains was separately tested in two groups of chickens plus a negative control group (only 0.2 ml of BHI injected/chicken).

* Bojesen et al. (2003). Liver isolate from Denmark.

** Edgardo Soriano, Tepatitlán Jal. México.

*** Moderate splenomegaly with petechial hemorrhages. dpi.- days post-infection. Rational numbers represent the proportion of affected between unaffected animals of each group.

### 3.4. Fimbrial mRNA quantitation in *Gallibacterium anatis* ESV200

**Figure 2:**
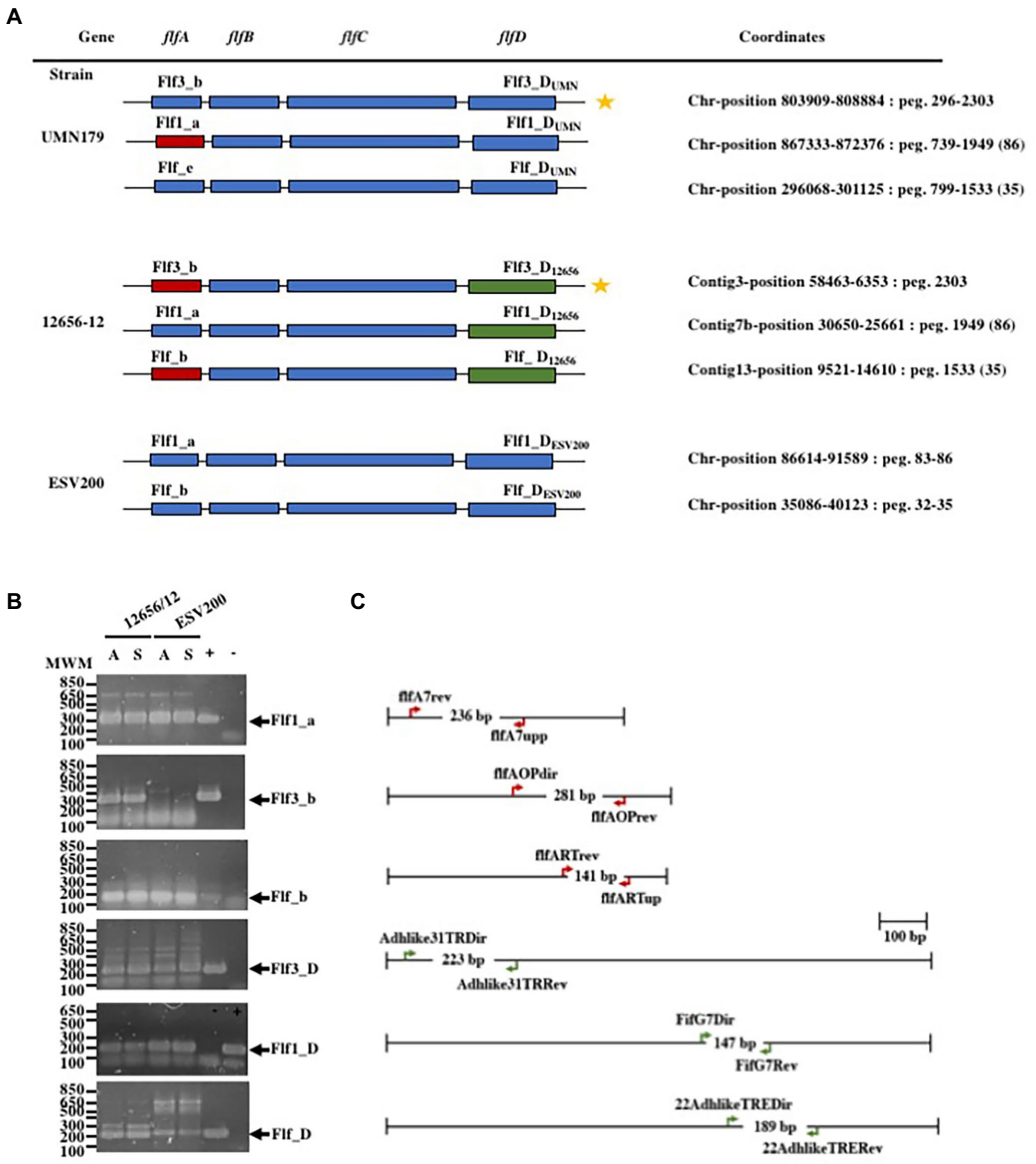
Gene organization in the F17-like operons from *G. anatis* and gene expression tests. **(A)** Gene arrangement in the three F17-like operons harbored in *G. anatis* UMN179 and 12656-12 strains, and from the two operons found on the genome of *G. anatis* ESV200 strain; The molecular classification of the genes into operons was made according to Kudirkiene et al. (2014), and the name operons are shown for each one; the coordinates for operons within each genome is also shown. Capital letters A, B, C or D were added to distinguish between the genes encoding the major protein, the first chaperone, the Usher chaperone and the adhesin. In subscript the name of the strain was added. **(B)** Amplified DNA fragments obtained by the qRT–PCR test of the expressed fimbrial and adhesin genes from *G. anatis* 12656-12 and ESV200 strains; because the Flf3_b fimbrial gene (star labeled) homologous to the present in *G. anatis* 12656-12 strain does not exist in *G. anatis* ESV200 strain, as well as the Flf_D gene, spurious cDNA amplification in low quantity, was observed. +/− Positive or negative reaction controls. **(C)** Diagrams of the indicated genes in A are shown to scale, and the oligonucleotide DNA primers used as well as the expected size of the amplified DNA product are shown.

**Figure 3:**
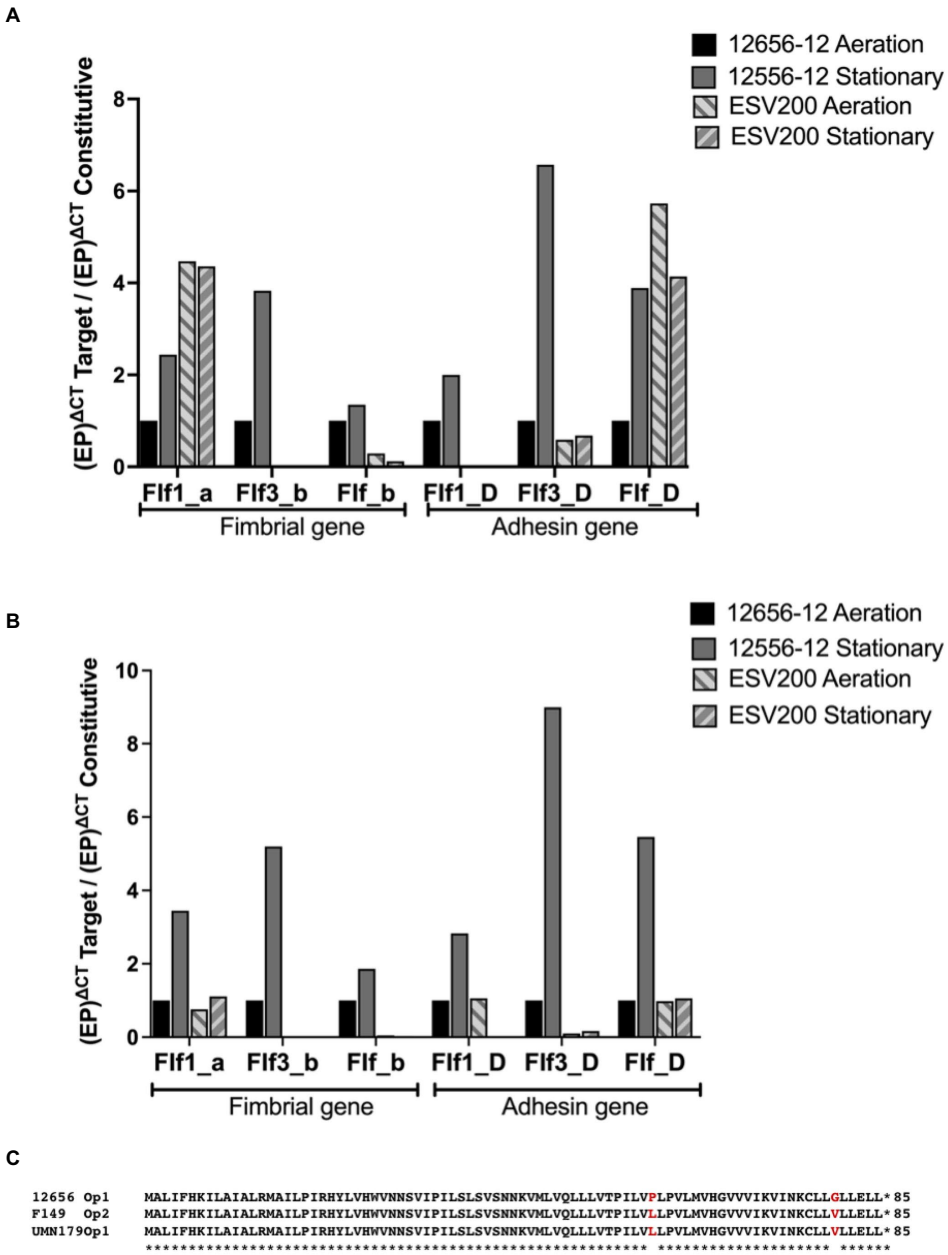
Inducible expression of F17-like genes in *G. anatis* 12656-12 strain and noninducible expression in *G. anatis* ESV200 strain. **(A)** The gene expression quantitation of mRNA from F17-like operon genes was achieved by qRT–PCR. In this essay, the comparison with the constitutive *rpo*A-gene encoding the RNA-polymerase alpha subunit was included. **(B)** The gene expression quantitation of F17-like genes was performed by comparing the expression of the gene encoding elongation factor G. The efficiency of the qRT-PCRs was greater than 96%, with an R^2^ ≥ 0.86. **(C)** Amino acid sequence deduced from the open reading frames identified in the genome of several *G. anatis* strains but absent in the genome from *G. anatis* ESV200 strain; this protein was not identified in any database consulted, such as GenBank, ExPASy, PDB, or by searching with BLASTp.

As changes in bacterial aggregation have been observed through treatment with proteases and fibrillar structures by electron microscopy of *G. anatis* biofilms (Vaca et al., 2011; Salgado Lucio et al., 2012), we asked how some extracellular proteins of *G. anatis* are expressed, as they could form cellular junctions within the biofilms or bridges of cells with solid surfaces, so the abundances of the mRNA of the fimbrial genes flf1_a, flf3_b, flf_b, flf1_D, flf3_D, and flf_D (Figure 2A) were determined at the transcriptional level (Figure 2B). Absolute quantitation of the transcripts by qRT–PCR was performed by using standard curves prepared for each of the genes; the expression of each transcript was different, and the flf3 gene primers did not amplify any fragment from ESV200 DNA, either due to variations in the target sequence of the primers or due to the absence of the gene in the genome of the ESV200 strain. Another gene that is poorly expressed in *G. anatis* ESV200 is flf_D. However, the structural and adhesin genes of the flf_b operon of ESV200 correspond to two different operons in *G. anatis* 12656-12; the homologous but more divergent adhesin (89% identity) is part of the flf3_b operon, and the remaining three genes, including the gene encoding the major fimbrial protein (98% identical), are present in flf_b. According to the expression analysis of the fimbrial genes in strain 12656-12, higher expression was induced under static conditions (Figure 3).

The relative expression of fimbrial mRNAs with respect to the expression of mRNA of the RNA polymerase gene (Figure 3A) showed a constant expression level in the 12656-12 strain under static conditions, and the expression levels of the three mRNAs of the three fimbrial genes (0.5 at 4X) and the three mRNAs encoding adhesins (1 to 7X) changed under agitation conditions.

For the ESV200 strain, 4X greater expression of the mRNA encoding the Flf1_a protein was observed, regardless of the culture conditions, without the expression of Flf1_D. For the major protein Flf3_b, there was no expression in the ESV200 strain, and for adhesin, low expression was observed, perhaps as an important housekeeping gene. For the major protein Flf_b in ESV200, vestigial expression was observed, while increased expression (4X) of Flf was observed. In a comparative analysis of expression of the target genes with that of the housekeeping gene encoding EFG (Figure 3B), basal expression was also observed under aeration conditions in the 12656-12 strain, while the expression of the fimbrial proteins increased from 2X to 5X under static conditions; the expression of adhesins increased from 3X to 9X. In strain ESV200, there were no important changes in the expression of the majority fimbrial protein or adhesin, although under aeration, low expression of the adhesin Flf_D was observed (Figure 3).

### 3.5. The ESV200 strain genome contains fimbrial genes

**Figure 4:**
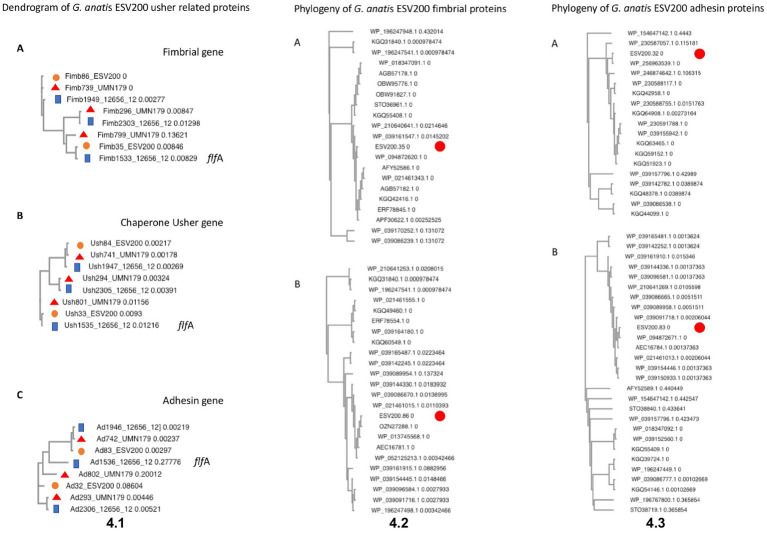
Molecular analysis of DNA and proteins encoded by *G. anatis* F17-like fimbrial operons. (4.1) Phylogeny of F17-like genes. Clusters were constructed by gene comparison of *G. anatis* sequences from usher containing operons with Clustal Omega (Madeira et al., 2022): the sequences included here were from *G. anatis* ESV200, UMN179, and 12656-12 strains. **(A)** The phylogram of fimbrial genes from three *G. anatis* strains. **(B)** The cluster of usher-chaperone genes from three *G. anatis* strains. **(C)** The cluster of adhesin genes from *G. antis*. The genes named here contain the GenBank database tags. Examples: fimbrial genes – Fim plus tag; usher genes – Ush plus tag; and adhesin genes-Ad plus tag. *flf*A- Reference *flfA* operon genes are indicated in the cladograms. The length of the branch is indicated in each sequence name. (4.2) Phylogeny of F17-like fimbrial proteins from the *G. anatis* ESV200 strain. The phylogenetic trees were reduced leaving the *G. anatis* closely related sequences and eliminating duplicated records from GenBank: **(A)** Tree with sequences closely related to fimbrial protein ESV200-tag-32; **(B)** Tree with sequences closely related to fimbrial protein ESV200-tag-83. (4.3) Phylogeny of F17-like adhesin proteins from the *G. anatis* ESV200 strain. The phylogenetic trees were reduced leaving the *G. anatis* closely related sequences and eliminating duplicated records from GenBank: **(A)** Tree with sequences closely related to adhesin protein ESV200-tag-35; **(B)** Tree with sequences closely related to adhesin protein ESV200-tag-86.

To further understand the form of expression of the fimbriae in *G. anatis* ESV200, the sequence of the ESV200 genome was obtained, and information on the fimbrial operons was extracted. This information was compared with that of the genomes of strains UMN179, 12656-12 and F149^T^. The ESV200 strain genome was found to contain two fimbrial operons, while the other three strains possessed three operons (Figure 2). These operons are based on a secretion system that involves the Usher chaperone located on the outer membrane of the bacterium. This chaperone is the largest protein of the chaperone/Usher system that, together with another smaller periplasmic chaperone encoded by the fimbrial operon, is involved in the secretion of the fimbrial structure. The sequence of the usher chaperone gene of the ESV200 strain was compared with others (Figure 4), and it was found that, in addition to the open reading frame (ORF) for the usher gene, there was an internal reading frame in one of the usher genes in strains 12656-12, F149^T^ and UMN179 (Figure 3C), but this reading frame was absent in the ESV200 strain. The absence of this extra ORF may be related to the lower biofilm production by this strain.

#### 3.5.1. ESV200 genome reconstruction and comparative genomic analysis

Initially, the genome of the ESV200 strain was assembled from 55 contigs containing 2.54 Mbp, which were obtained from reads from Illumina and 454 technologies after filtering for sequence quality with FastQC and applying Velvet software to reconstitute the genome into contigs. A circular genome was obtained with long reads from Sequel PacBio technology and assembled with Spades and Canu software. The ESV200 genome was reconstructed *de novo* with 32X coverage and N50 = 2,538,577. A full-length revision was made with RAST and deposited in the GenBank database with accession number CP114281.1 for final annotation with the NCBI Prokaryotic Genome Annotation Pipeline. The genome has a 40% GC content and contains 7, 6 and 6 copies of the 5S, 16S and 23S rRNA genes, respectively, and 61 tRNA copies. The Gtx-toxin operon was found to share 94% identity with the nucleotide sequence (HQ316167), and GtxA shared 100% identity with WP_094873868.1 and 99% identity with toxin proteins from the UNM179 and 12656-12 strains. Additionally, two chaperone/usher fimbrial operons were found (see above, Figures 2, 4). There were 3 CRISPR arrays and 71 mobile element proteins present. To date, 39 genomes of this species have been reported, and only UMN179 has 1 circular genome with 39.9% GC content, 2.68 Mbp, and 7, 6, and 6 copies of the 5S, 16S and 23S rRNA genes, respectively, making this strain a high-confidence reference for exploring the core genes of the ESV200 strain. The genetic core of the 4 genomes consisted of 2,061 correctly annotated genes with known functions, and hypothetical genes were discarded. Table 3 shows the set of genes shared between genome-strain pairs selected from GenBank: AVOX00000000.1, UGGZ00000000.1, CP114281.1, and CP002667.1. The comparison provides useful information about common genes and other strain-specific genes, for example, the genes for fimbrial attachment and colonization of host tissues mentioned above (Figure 4).

**Table 3:** *Gallibacterium anatis* ESV200 genome comparison.

Strains*	12656-12	NCTC11413	ESV200	UMN179
12656-12	2337 **	-	-	-
NCTC11413	2,343	2,256	-	-
ESV200	2,341	2,297	2,315	-
UNM179	2,359	2,322	2,326	2,324

* Four GenBank genomes from *G. anatis* strains were compared by pairs; the selected genomes in this analysis were those containing the longest genome size or few contigs.

** Matrix values indicate the total number of homologous genes contained between strains. The values in the diagonal direction indicate the total gene number from each strain reported in the GenBank database. The table shows the number of genes shared between strains in a pairwise comparison. The hyphen symbol is included to avoid duplicating values in pairwise comparison. The number of probable genes shown is derived from the comparison between genomes using the RAST platform. Numerical differences are due to gene duplications or cross-homologous sequences containing multiple domains.

**Figure 5:**
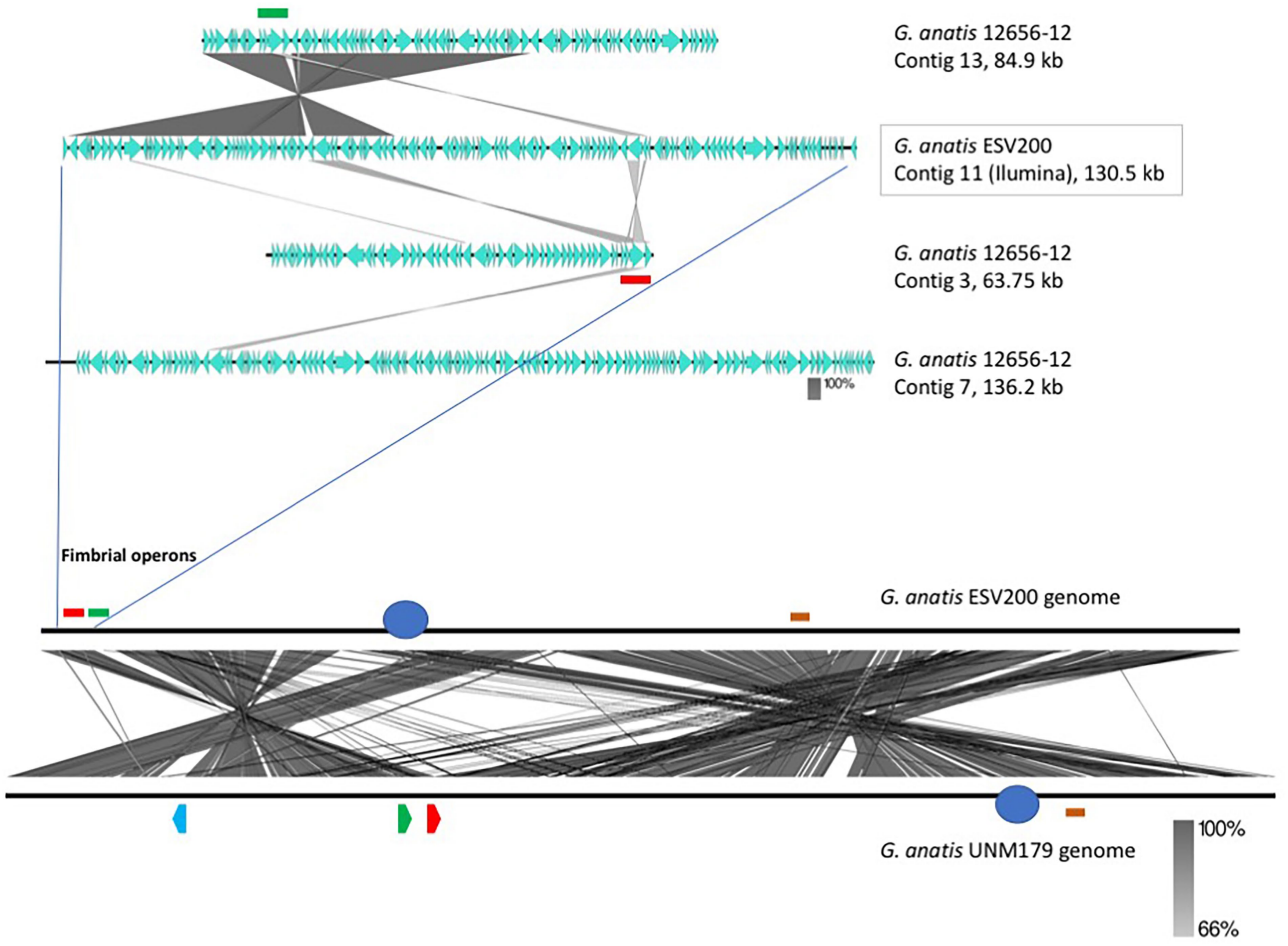
Identification and localization of F17-like fimbrial operons harbored in the *G. anatis* ESV200 genome. **(A)** Identification and localization of F17-like fimbrial operons harbored in *G. anatis* ESV200 and 12656-12 strains. The *G. anatis* fimbrial operons containing the usher-chaperone gene were identified by BLASTn and BLASTx (www.ncbi.nlm.nih.gov; Madden et al., 1996) and Clustal Omega (www.ebi.ac.uk/Tools/msa/clustalo/) programs. The image shows the identity regions between 130,591-base-long contig 11 from the *G. anatis* ESV200 strain and three contigs (No. 3, 7, and 13) from the *G. anatis* 12656-12 strain. Contig 11 from the *G. anatis* ESV200 strain has a large region with identity toward contig 13 from the *G. anatis* 12656-12 strain and harbors two fimbrial operons (highlighted with green and red colors). Each *G. anatis* 12656-12 contig harbor only one fimbrial operon. **(B)** Full alignment of *G. anatis* ESV200 and UMN179 genomes. The ESV200 genome was reconstructed through *de novo* sequencing using Sequel PacBio technology with 32X coverage. Two black horizontal lines show the comparison along the two *G. anatis* genomes, and the crossed lines between the two parallels indicate mainly stretches of homologous sequences. The crossed lines are the result of hundreds of direct and inverted matches, and here are observed two notorious regions for being inverted. The two sections are approximately equivalent to 30 and 60% of the compared genomes. Image obtained with Easyfig software.

**Figure 6:**
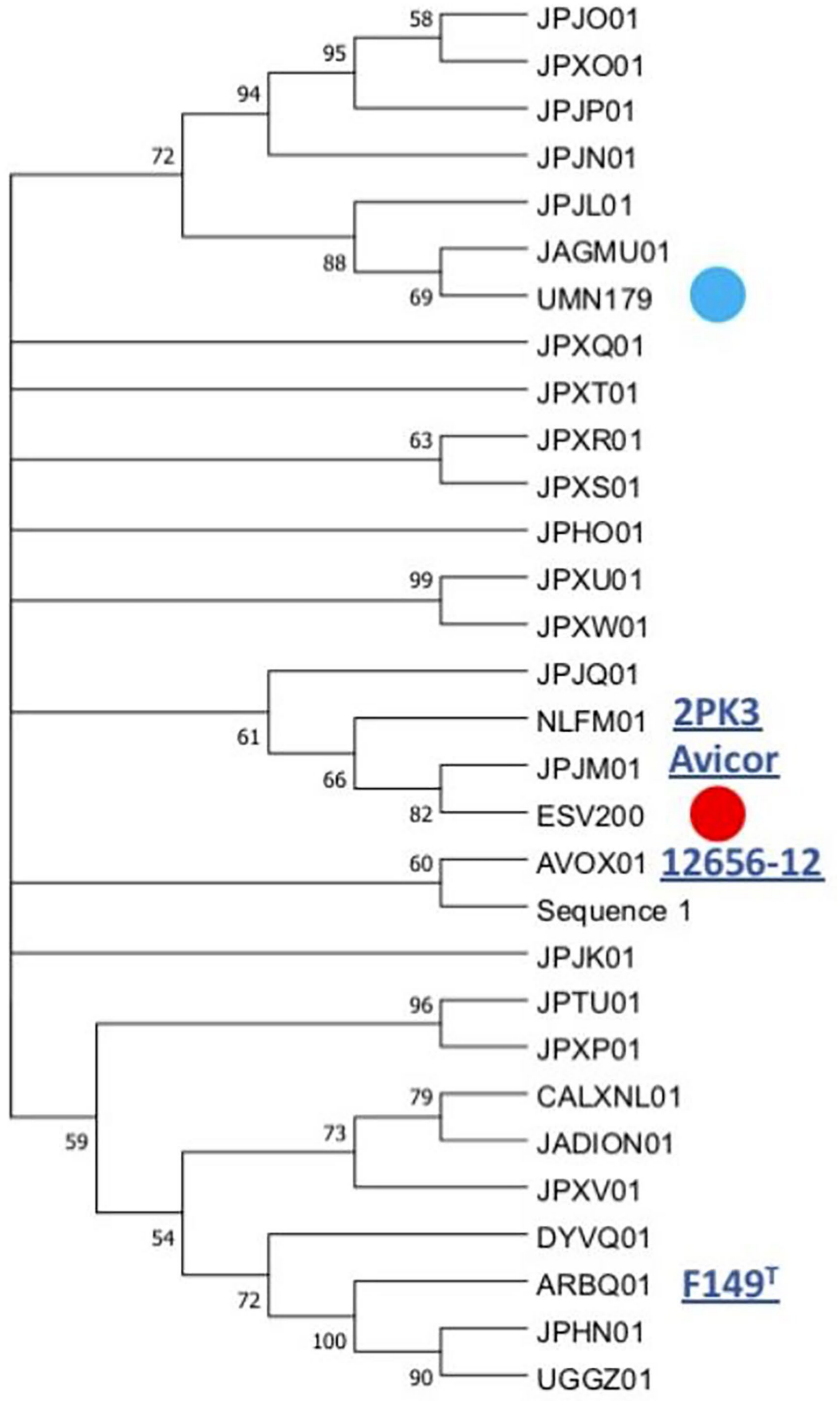
Phylogenetic grouping of the *G. anatis* ESV200 strain by MLST. The analysis was performed using the next concatenated sequences harbored in thirty-one *G. anatis* genomes: *adk, atp*D, *fum*D, *gyr*B, *inf*2, *mdh*, *rec*N, and *tdh*. This phylogram was obtained by Kalign (Lassmann, 2019) and iTol (Letunic and Bork, 2021) online programs. Here, *G. anatis* MLST sequences form a very compact group, although the *G. anatis* ESV200 sequences are closer to the *G. anatis* 2PK3 and Avicor sequences. The names in the phylogeny tree are the accession number prefixes used in GenBank except ESV200 and UMN179 to easily identify them. Four names of the reference *G. anatis* strain are also highlighted on the outside of the tree. The evolutionary history was inferred using the maximum parsimony method. The most parsimonious tree with a length of 3,105 is shown. The consistency index was 0.423833 (0.348744), the retention index was 0.509460 (0.509640) and the composite index was 0.215926 (0.177671) for all sites and parsimony-informative sites (in parentheses). The MP tree was obtained using the Subtree-Pruning-Regrafting (SPR) algorithm with search level 1 in which the initial trees were obtained by randomly adding sequences (10 replicates). This analysis involved 30 nucleotide sequences. A total of 12,441 positions was included in the final dataset. The evolutionary analysis was conducted using MEGA v.11 software. Supplementary material contains sequence information, accession numbers, and genomic sequence tags.

The linear comparison of the genomes of the strains ESV200 and UMN179 showed a significant number of chromosomal rearrangements but maintenance of the functionality responsible for pathogenicity in birds (Figure 5). The phylogenetic comparison of the strains that have had their genome sequenced to date, by using a group of genes suggested to be MLST markers, showed interesting groupings, although there was not much distinction among the sequenced strains, at least with this method (Figure 6). The evolutionary history was inferred using the maximum parsimony method. The most parsimonious tree with a length of 3,105 is shown. The consistency index was 0.423833 (0.348744), the retention index was 0.509460 (0.509640) and the composite index was 0.215926 (0.177671) for all sites and parsimony-informative sites (in parentheses). The MP tree was obtained using the Subtree-Pruning-Regrafting (SPR) algorithm with search level 1 in which the initial trees were obtained by randomly adding sequences (10 replicates). This analysis involved 30 nucleotide sequences. A total of 12,441 positions was included in the final dataset. The evolutionary analysis was conducted using MEGA 11 software.

### 3.6. Integrases in the genome of *Gallibacterium anatis* ESV200

Nine integrase-related amino acid sequences, ranging in size from 60 to 415 amino acids, were deduced *in silico* from the *G. anatis* ESV200 genome. A 168 amino acid sequence was annotated as an integrating regulator; therefore, the regulator size selection criterion was used to separate the integrase sequences into two categories. Thus, five large sequences remained as integrases (252 to 415 aa) and the four short ones as possible integration regulators (60 to 170 aa). The short sequences showed a conserved amino-terminal domain of 60 amino acids, although the integrase regulator had more significant differences from the other short proteins. Essential amino acids that are part of the active site for integration, including tyrosine, were identified in proteins from 252 to 415 aa. Three amino acid sequences related to integrases delimited a 539.6 kb fragment. This sequence is flanked at the ends by two segments of mobile genetic elements, at one end an episome type and at the other a prophage element, with 50.5 and 46.3 kb, respectively. The remarkable aspect of the chromosome fragment is that it contains chromosome, phage, and plasmid replication gene clusters. Mobilization genes and plasmid transfer. Genes that can provide resistance to antimicrobial substances as ejection pumps. Genes for RNA polymerase peptide biosynthesis. Genes involved in ribosome biosynthesis, such as ribosomal RNA and ribosomal proteins. Genes for the biosynthesis of aminoacyl-tRNA synthetases. Genes for the biosynthesis of chaperones for the export of extracellular proteins. Genes for fimbriae biosynthesis and control of biofilm formation. By size, this segment resembles a bacterial minichromosome. However, due to the lack of essential genes, it has an integron-type structure, similar to those called Maverick. This chromosome segment is equivalent to a domain called “D1,” composed of 22% of all genes. The “D2 and D3” domains, delimited by the mobile element regions, contain 25.2 and 52.8% of all genes, respectively. The D1 domain contains 5% hypothetical and DNA mobility genes. Regarding the exclusive DNA mobility genes, D1 contained 3.1%, D2 6.1%, and D3 0.94%. This means more significant potential for DNA mobility in the small domains than in the large domain of the *G. anatis* ESV200 chromosome.

## 4. Discussion

Several diseases in birds are caused by *G. anatis* and are generically known as gallibacteriosis. Biofilm formation requires the establishment of a colony on superficial tissues and, if conditions allow it, on internal surfaces or internal organs; for this, the interaction of bacterial adhesins with host receptors is essential. These colonization processes can be very beneficial for the bacterium under antimicrobial treatment, as bacteria exhibit resistance in biofilms, which can be formed by several pathogens, including those of the *Gallibacterium* genus. It has also been reported that fimbriae are important for the formation of biofilms and that they are even targets of vaccination therapy *via* the development of fimbrial protein antigens. Here, we examined the presence of genes that encode fimbrial proteins and their transcriptional expression in the ESV200 strain, a pathogenic strain that apparently produces little biofilm *in vitro*.

In this work, the biofilm formation phenotype of strain ESV200 was compared with that of strain 12656-12, a bacterium included in several previous reports. The ESV200 strain forms highly homogeneous suspensions in liquid cultures, while the 12656-12 strain forms clumpy cultures that can settle rapidly when left under static conditions for a short time. However, biofilm quantitation performed on cell culture plates showed that the ESV200 strain produced as much biofilm as the 12656-12 strain at 24 and 48 h, but this production was reduced after 72 h. This behavior could be attributed to the change in the expression of genetic regulators that are still unknown under laboratory conditions or to loss of viability. The strain 12656-12 showed maintenance of the biofilm structure over time. Under such conditions, it would be expected that there would be differences in virulence if the aggregation conditions and biofilm formation were highly relevant in the pathogenic process due to better adhesion to host tissues (Salgado Lucio et al., 2012).

The pathogenicity tests showed a similar pattern of damage produced by the two strains tested, with heart damage caused by strain 12656-12 but not by ESV200. We could not confirm that the lower quantity of biofilm produced at 72 h by the ESV200 strain was the cause of the lack of heart damage, since molecular components of the genetic background that have not been evaluated may have played a role; for example, the *in silico* analysis indicated that the biosynthesis of cardiolipins that are present in both strains might indicate the surface compatibility of the bacterium with cardiac tissue (Rossi et al., 2017). The genomes of the ESV200 and 12656-12 strains contain two genes homologous to those involved in the pathogenicity of *Salmonella enterica*, *pbg*A and *ics*A (Chiu et al., 2005; Cian et al., 2019).

The biofilm produced by *G. anatis* was altered by the biofilm-disrupting treatment conditions (Figure 1C). DNase and proteinase K altered the ESV200 strain biofilm, as indicated by the increase in turbidity, indicating the participation of DNA and proteins in biofilm structure formation and cell attachment. Disintegration was also observed for the 12656-12 strain biofilm treated with DNase. The reduction in turbidity may also have been due to extensive activity of the pronase E and proteinase K enzymes; therefore, the presence of proteins in the biofilm of *G. anatis* was explored. The proteins were revealed by electrophoretic separation of the total extracts of both strains and analyzed, and the sizes ranged from >10 to <75 kDa; the sizes of fimbrial proteins ranged from 22 to 43 kDa. Lysozyme and urea further improved the protein resolution in the SDS–PAGE system, which may be because some proteins became free in solution and by inhibition of proteolytic activity (Tamura et al., 1998; Garcia-Gomez et al., 2005).

Regarding the possibility of the biosynthesis of fimbriae of the F17 type proposed by Bojesen, two fimbrial operons were identified in the genome of the ESV200 strain, which corresponded to the chaperone/usher secretion system, one of which showed 99% conservation with the gene present in the 12656-12 strain, while the other corresponded to a secretion system, exhibiting 98, 92 and 97% identity with the major fimbrial protein, the chaperone and Usher, respectively, whereas the putative adhesin exhibited 89% identity with the third operon of the 12656-12 strain, which suggests that an important recombination process led to the deletion of an operon in the ESV200 strain and the exchange of genes as modules within the fimbrial operons, and perhaps in this way, it gains greater genetic and functional variability, as occurs with other microorganisms (Dufresne et al., 2017). This conclusion was made by sequencing the genome of strain ESV200, which made it possible to clarify why not all the transcripts present in strain 12656-12, evaluated at the beginning of the investigation, could be quantified. Investigation of the identities of the adhesins showed that the adhesin FlfA shared 99% identity with that present in the 12656-12 strain, 30% with the *E. coli* adhesin TPA (HAN7666271.1) and 29% with the *S. enterica* protein (EDB7133990.1), while the adhesin FlfA7 shared 88% identity with the protein from 12656-12, 36% with the *E. coli* adhesin F17 (STJ01015.1) and 32% with F17G (EFA5996531.1). The adhesin absent in the ESV200 strain but present in the 12656-12 strain genome shared 33% identity with the *E. coli* adhesin TPA (HAN7666271.1) and 31.6% identity with the *S. enterica* adhesin (EAM2645026.1).

The presence of several F17-type operons varies among *G. anatis* strains, since more than one fimbrial operon may be present and since some of them have mutations that change them into pseudogenes, so it is expected that the content and type of fimbriae will depend on the type of strain tested (Johnson et al., 2013; Pors et al., 2016). In the ESV200 strain, the two operons seem to express themselves with different transcription rates according to the estimates made with two housekeeping genes used in this work, namely, RNA polymerase and elongation factor G. Interestingly, it was observed that the ESV200 Flf_a mRNA was strongly expressed under static conditions and agitation, similar to the adhesin Flf. For the mRNAs of the fimbrial proteins and adhesins of the 12656-12 strain, notable increases in expression levels of 1x to 6x more than the transcription of the RNA polymerase subunit were observed for all the operons in the static condition, perhaps due to the distribution of molecular regulators. Therefore, the immunity strategies based on the fimbrial proteins proposed by Bojesen are functional in strains that exhibit the expression behavior seen in strain 12656-12 or in strains that have different types of fimbrial operons, which seem to be conserved in *G. anatis* for their importance in the colonization of avian tissues (Bojesen et al., 2004; Salgado Lucio et al., 2012; Pors et al., 2016).

It is currently important to obtain a new fully sequenced and circularized *G. anatis* genome to accurately compare the possible presence or absence of genes that are frequently detected as multiple copies, whether they are involved in basic functions such as those of the biosynthetic machinery or other functions related to virulence, such as colonization.

**Figure 7:**
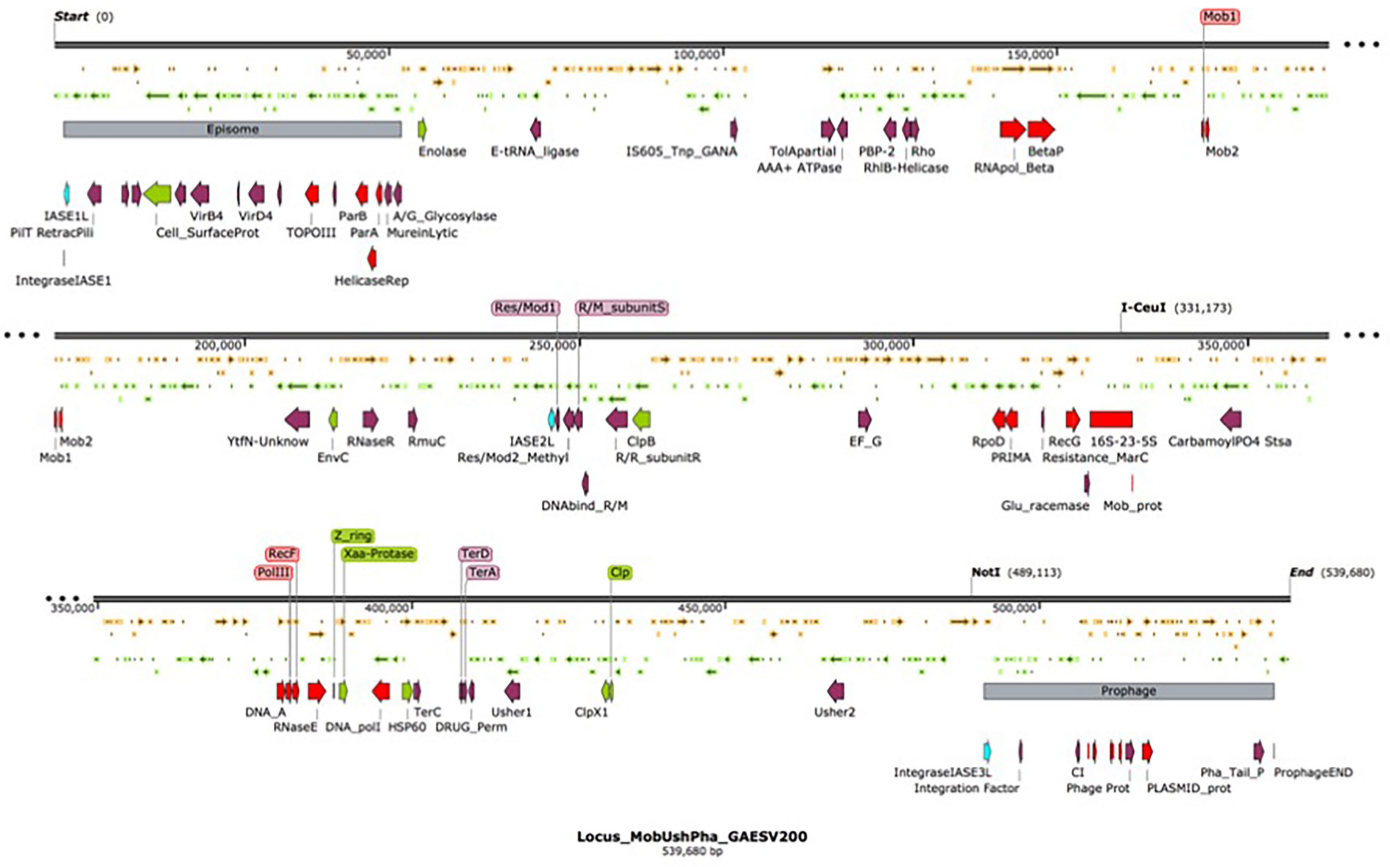
Organization of the chromosomal domain D1 of *G. anatis* ESV 200 displaying a complex Maverick-like structure. Domain D1 of the three chromosomal domains of *G. anatis* ESV200 is shown here. The domains are separated based on the presence of large mobile genetic elements, the D1 domain is composed of 539.6 kb, and 520 ORFs have been deduced. The D1 domain contains an episome and a prophage at the ends, and there are also open reading frames encoding putative integrases. Some sequences that code for proteins related to DNA mobility, integral DNA maintenance, recombination, host colonization, participation in the control of biofilm formation, drug resistance and cell division have been highlighted.

Location of the fimbrial operons in the genome of *G. anatis* ESV200. It has been documented that bacteria can fix their genome to perform better in the environment where they live, be it natural or laboratory (Nagy-Staron et al., 2021; Saleski et al., 2021). It has been proposed that genes in the genome have different levels of expression depending on their location and their syntenic organization, which in many bacteria has allowed the correlation of gene clusters with the virulence abilities of pathogenic bacteria (Le Berre et al., 2022). One of the outstanding aspects at the moment in the genetics of bacteria is the presence of islands of pathogenicity (Desvaux et al., 2020), which are short segments of the genetic material that concentrate genes that encode virulence factors (Wu, 2015), antibiotic resistance functions (Debroas and Siguret, 2019; Ellabaan et al., 2021; Maciel-Guerra et al., 2022), proteins that ensure DNA perpetuation, and enzymes involved in the internal mobility of DNA (Hallstrom and McCormick, 2015; Smyshlyaev et al., 2021). Due to their functional structure, they can be contained in plasmids, transposons, integrons, or phages (Dobrindt et al., 2015). In the genome of bacteria of the *Pasteurellaceae* family, the presence of a significant number of sequences related to DNA mobility, such as integrases or transposases, has been observed (Roodt et al., 2012; Horta-Valerdi et al., 2017). In the genome of *G. anatis* ESV200, together with the Usher operons that encode the fimbriae, an insertion sequence was found that could represent an integron, similar to pathogenicity islands (Carniel et al., 1996; Mazel, 2006). When inspecting toward the ends of the operons, two insertion sequences were located, one next to 50.5 kb of an episome and another next to 46.3 kb of a prophage. The segment or D1-domain delimited by both mobile elements is approximately 539.6 kb in size (Figure 7). The inspection of this segment of DNA concentrates most of the vital functions for the cell, DNA Primase, DNA polymerase, components of the main RNA polymerase, various recombinases, antitoxin-toxin system, DNA translocation, transfer of genetic material, production of fimbriae, general mechanisms of resistance to antibiotics, genes responsible for the synthesis of ribosomal RNA 16–23-5S and control of biofilm formation. This large sequence resembles the mobile structures of eukaryotes called Maverick, associated with c-integrase, and in size, they almost reach the size of a *Mycoplasma* genome (Suthers et al., 2009).

## 5. Conclusion

The genome of *G. anatis* strain ESV200 is the second genome of this species to be completely sequenced since 2011, with an estimated genome size of 2,538,577 nucleotides. With a group of classic MLST genes, a phylogenomic association can be achieved, although with another group of genes, other close evolutionary relationships between *G. anatis* strains can be observed, which is always interesting when characterizing new isolates to differentiate them from some clonal types.

The ESV200 strain is virulent in birds and produces fimbriae related to colonization and virulence; it contains several virulence genes, one of which encodes the toxin GtxA, the main virulence factor of *G. anatis*. Shown here is the structure of a chromosomal domain, which is armed in a way recently described, named Maverick, and which can impact the evolution of pathogens, such as *G. anatis.*

Strain ESV200 can be used as a molecular model for gene knockout studies as well as for genetic complementation because it naturally contains only two Usher-type fimbrial operons and exhibits a weaker clumping phenotype in biofilms.

## Data availability statement

The datasets presented in this study can be found in online repositories. The names of the repository/repositories and accession number(s) can be found in the article/Supplementary material.

## Author contributions

EC-J sequencing DNA, assembling genomes, and DNA analysis. MA-R and LL-R biofilm assays and analysis. GP-C and FV-P sequencing DNA by Ilumina technology and special reacts. EA-M and AL-O qRT-PCR development and analysis. VP chicken bioassays and analysis. PS-A, EN-A, and CV-C wrote projects to get financial resources, design the project, and write and main review of the manuscript. All authors contributed to the article and approved the submitted version.

## Funding

We are grateful to CONACYT México for the fellowship No. 297565 for Cobos Justo ME, to CONACYT México for project CB-2015-01-259209, BUAP-VIEP-00265, BUAP-VIEP-100103133-2022, and UNAM-DGAPA PAPIIT IN204122 and to Edgardo Soriano for the kind donation of the bacterial ESV200 strain used in this study.

## Conflict of interest

The authors declare that the research was conducted in the absence of any commercial or financial relationships that could be construed as a potential conflict of interest.

## Publisher’s note

All claims expressed in this article are solely those of the authors and do not necessarily represent those of their affiliated organizations, or those of the publisher, the editors and the reviewers. Any product that may be evaluated in this article, or claim that may be made by its manufacturer, is not guaranteed or endorsed by the publisher.

## Supplementary material

The Supplementary material for this article can be found online at: https://www.frontiersin.org/articles/10.3389/fmicb.2023.1084766/full#supplementary-material

## Footnotes

^1^ https://www.ebi.ac.uk/ena/data/view/GCA_000464615.2

^2^ www.ebi.ac.uk/Tools/msa/clustalo/
