# Supplementary figures and images for: A Maverick-like cluster in the genome of a pathogenic, moderately virulent strain of Gallibacterium anatis, ESV200, a transient biofilm producer
Source: Front Microbiol. 2023 Jan 26;14:1084766. doi: 10.3389/fmicb.2023.1084766 (PMC9909271; doi:10.3389/fmicb.2023.1084766)

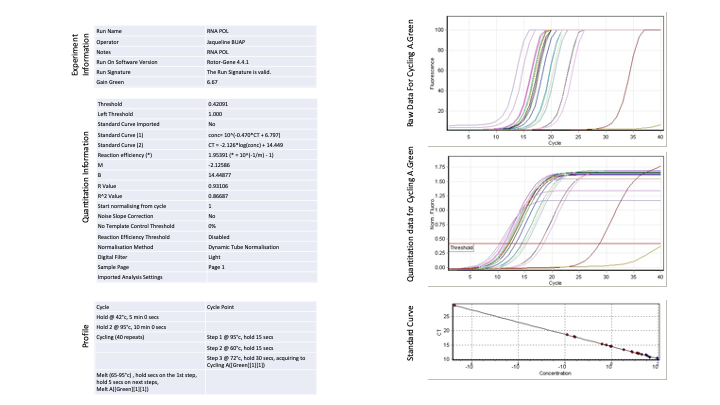

Supplement: Supplementary file 4 [file Image_1.png]

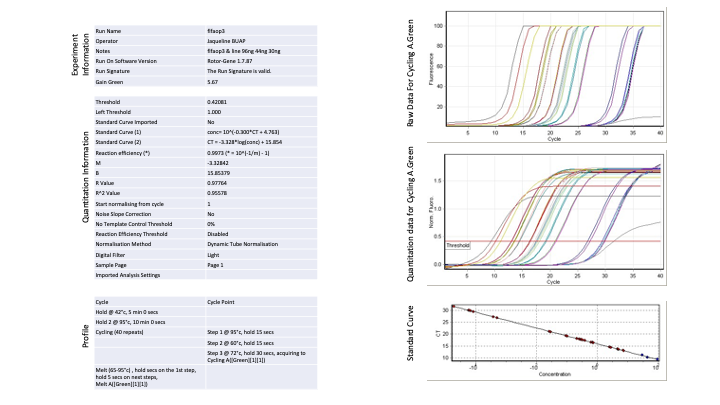

Supplement: Supplementary file 5 [file Image_2.png]
